# Joy at work: how to build a happy and resilient next generation of radiologists

**DOI:** 10.1007/s00330-023-10146-9

**Published:** 2023-08-25

**Authors:** Isabel Molwitz

**Affiliations:** Department of Diagnostic and Interventional Radiology and Nuclear Medicine, University Medical Center Hamburg-Eppendorf, Martinistraße 52, 20246 Hamburg, Germany

Happiness and resilience are key to ensuring the power of the radiologists’ workforce in the future. Besides the moral side of supporting employee happiness, happiness and resilience are vital in times of staff shortage.

In fact, the already existing shortage of radiologists and radiographers is continuously aggravated by increasing workload [1]. In addition, there is an increasing desire for part-time work and work-life balance. Furthermore, the number of female radiologists is growing, and women still regularly work part-time due to gender imbalances in family care workloads and insufficient childcare infrastructure in many European countries. Under this premise, burnout rates among radiologists, which have been reported with up to 50% [2], are alarming. That across cultures already among residents high rates of burnout symptoms have been observed [3, 4] must force us to reflect on how we can ensure resilience among the next generation of radiologists.

But let’s be honest, resilience is not enough. As some colleagues have recently stated: “If the goal is to address burnout, reduce the high suicide rate, and combat depression then the work site is NOT a place where people look forward to gathering.” [5]. We do not simply want to keep radiologists from becoming depressed and burned out. What we should strive for and what we need is to excite and win bright, engaged young colleagues for our specialty. Happiness and joy at work do not only augment productivity but also reduce turnover rates, and correlate with lower costs and organizational growth [6]. It will be with a happy next generation of radiologists that we can grow further, enlarge the radiological portfolio, educate our students, contribute to cutting-edge research, and offer our patients the best available diagnostics and interventional therapies.

Let’s start with happiness. How do we build a happy next generation of radiologists? Luckily, most radiologists already judge their profession as meaningful and have joy in what they do [7]. Besides the expectation of joy at work, in a recent survey among > 500 radiologists of all career levels from Germany, high rates of agreement were found for expectations of a good working atmosphere, support for further qualification, a structured and timely residency, good income, reliable working times, family friendliness, career development, and opportunities to shape the work environment [7]. Interestingly, a good income was the least important criterium for younger radiologists (even if still 73% expected it), while family friendliness and reliable working time were particularly important for all participants below the chief physician position [7]. Most of these wishes seem realizable by structured processes and protected time slots.

If structured schedules with a punctual ending of the working day or flextime work were department standard, radiologists with children—especially women—would feel less emotional strain, themselves and from the team, about “leaving early”. They would be full team members and could focus on pursuing their careers, likely a situation that would increase their happiness. Also, we would no longer force team members to choose between work-life balance and their career, a choice which the career and thus radiology may not win.

A structured training schedule during residency and for continued education, as well as sufficient time for all rotations including angiography, would help to meet corresponding expectations [8, 9]. Structured training curricula on European [10] and national levels [11] already exist. Local courses, e.g., regular case discussions once a week, could be organized correspondingly. Especially in small departments, structured curriculum-based digital courses may be helpful, which in part are even available for free [12]. Also, angiography simulators or virtual reality simulations allow the early practice of complex interventions [13]. However, those are often cost-intensive. A solution may be shared purchases or open classes for residents from smaller hospitals nearby.

Furthermore, concepts of happiness include mindfulness. In the work context, this refers to focusing on one task at a time. In radiology, where a lot of interdisciplinary communication takes place, this means protected time for reporting. A simple way for doing so is by separating workplaces that require a lot of communication, like, e.g., calls for CT indications, from reporting or interventions.

Lastly, free softdrinks or reduced sports and vacation offerings, as common in economics, would likely also be appreciated by physicians, even if not the main drivers of happiness at work.

And what about resilience? Resilience has been defined as the ability to respond to shock, injury, or stress in a healthy way [14]. It has multiple dimensions, for which not only individual but also institutional resources are important, such as support, teamwork, and institutional culture [14].

Again, it is much about how we consider and handle our time. Building a resilient next generation of radiologists requires a change of attitude. Traditionally, in medicine the longer you work, the more exhausted you are (and look), the more you have cared for your patients, and the better doctor you are. Evidently, this is flawed, and changes have been made to, e.g., shorten times on duty. It should become the new normal, that what we do with our time and not how long it takes us is what matters. If we teach that attitude to our students instead of taking pride in working over hours, they will likely be more apt to protect themselves and more resilient from the beginning. That it is important to teach the relevance and measures of increasing personal resilience early on is also confirmed by a recent study of 3666 Chinese radiological residents. In that study, those with higher levels of personal resilience were less prone to depressive symptoms when exposed to long working hours and night shifts [4].

Furthermore, in university hospitals, we need protected time slots for research. As supervisors, it is our responsibility to ensure the work quality of younger colleagues. Besides reports, this includes research. Due to the continuously increasing workload, free slots for research during the workday have dramatically decreased, while the international research competition increases. This entails a risk of low-quality research projects: ones that can still be planned and conducted with a sleepy brain after a long workday. Furthermore, in the current system, the most motivated young colleagues end up with the worst work-life balance. But work-life balance has been found to be an important element of physician resilience, helping with self-care, and countering stress and burnout [14]. If we want the next generation of radiologists to pursue high-quality research, while remaining healthy, we need to provide them with protected research time, e.g., by investing in grant writing and building research infrastructure (e.g., scan slots for study purposes in clinical routine).

Another important dimension of resilience is having control over the professional life [14]. Young radiologists expect opportunities to shape their work environment [7]. This is less an astonishing feature of the young generation but rather a known psychological need also for intrinsic motivation as described by the self-determination theory of Deci and Ryan [15]. Lower hierarchies, the inclusion of young colleagues into decision-making processes, and an active feedback culture increase motivation and the team spirit as an important institutional resource of resilience. Finally, workload as a main stressor in the first place could be reduced by digitalized processes and professional support with the increasing load of bureaucracy.

In conclusion, happiness and resilience are both important for the major goal of creating productive, happy, and healthy radiologists. There are multiple approaches how to enhance happiness and resilience, many related to well-structured processes, protected time slots, and changes in attitude. By building a happy and resilient next generation of radiologists, we can all contribute to a successful future of radiology.

## Declarations

### Guarantor

The scientific guarantor of this publication is Isabel Molwitz.

### Statistics and biometry

No complex statistical methods were necessary for this article.

### Methodology

• Commentary
